# Poldip2 takes a central role in metabolic reprograming

**DOI:** 10.18632/oncoscience.419

**Published:** 2018-06-23

**Authors:** Felipe Paredes, Izabela Suster, Alejandra San Martin

**Affiliations:** Division of Cardiology, Department of Medicine, Emory University, 308 WMB, Atlanta, GA, 30322, USA.

**Keywords:** lipoylation, poldip2, TCA, hypoxia, cancer

In 1951 Lester Reed isolated the first crystals of an “acetate-replacing factor”, appropriately named for its ability to replace acetate during the growth of lactic acid bacteria [1]; it was renamed α-lipoic acid (ALA) by Esmond Snell. After decades of research, we now recognize that ALA is a cyclic disulfide containing fatty acid that undergoes reversible redox ring opening/closing, which is coupled with the oxidative decarboxylation of α-keto acids. Thus, this essential prosthetic group occupies a critical position in energy metabolism by regulating the activity of the tricarboxylic acid (TCA) cycle.

It is well appreciated that metabolic plasticity is essential for cellular adaptation to physiological fluctuations in nutrient and oxygen supply. This adaptation, which benefits highly proliferative cells and contributes to tumor survival, relies on rapid activation and inhibition of biochemical pathways and notably, requires efficient communication between glycolysis, fatty acid oxidation (FAO) and the TCA cycle.

The pyruvate and the α-ketoglutarate dehydrogenase complexes (PDH and α-KGDH, respectively) sit at the center of mitochondrial catabolism. It has been clearly established that oxygen deprivation directs cells to restrain mitochondrial respiration by the action of hypoxia inducible factors (HIFs)[2]. Indeed, HIF-1α-mediated transcription of pyruvate dehydrogenase kinase 1 (PDK1) inhibits the PDH complex by phosphorylation and consequently blocks the synthesis of pyruvate-derived acetyl-CoA, the main substrate of the TCA cycle. However, this model does not predict inhibition of the activity of TCA enzymes and theoretically, cells under hypoxic conditions are still able to oxidize acetyl-CoA from non-glycolytic sources.

Importantly, PDH and α-KGDH are among the small group of four mammalian enzymes that utilize ALA as a catalytic cofactor. Interestingly, the lipoylation mechanism of these enzymes in mammals differs from those previously described in bacteria and yeast, and displays remarkable plasticity [3]. The basis of this plasticity relays on the utilization of a unique set of enzymes in a signaling pathway that includes the polymerase delta-interacting protein 2 (Poldip2).

**Figure 1:**
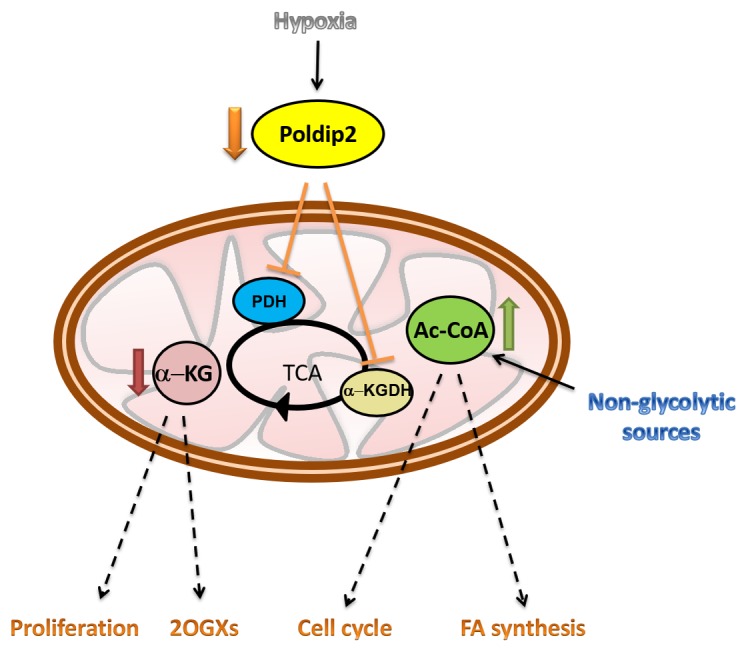
Poldip2 downregulation under hypoxia inhibits the lipoylation and activity of Pyruvate dehydrogenase (PDH) and alpha-ketoglutarate dehydrogenase (α-KGDH) As a consequence of tricarboxylic acid (TCA) cycle inhibition, α-KG is reduced while acetyl CoA is accumulated modifying downstream signaling pathways. 2OGXs: 2-oxoglutarate-dependent oxygenases; FA: fatty acids.

The expression of this nuclear-encoded, mitochondrial protein Poldip2 is essential for protein lipoylation of the PDH and α-KGDH complexes because it regulates the degradation of the lipoic activating enzyme, a key enzyme of the mammalian pathway. Poldip2 expression is rapidly inhibited under hypoxic conditions and basally repressed in highly glycolytic cancer cells [3]. In Poldip2 deficient cells, reduced protein lipoylation results in inhibition of the PDH and α-KGDH complexes and repressed mitochondrial function by restraining the entrance of pyruvate-derived acety-CoA and by inhibition of TCA cycle activity (Figure 1).

Due its unique ability to regulate the TCA cycle, downregulation of Poldip2 under hypoxia, not only impairs the utilization of acetyl-CoA derived from pyruvate but also from non-glycolytic sources such as FAO. Although the relevance of FAO in cancer cells has not been examined thoroughly, recent research has pointed to a crucial role of FAO as an essential source of reducing power for biomass synthesis and ATP. This could be particularly relevant for certain advanced solid tumors in which the utilization of fatty acids as a source of energy is increased [4].

Furthermore, inhibition of the TCA cycle may have two major consequences. First, the alteration of intracellular concentration of TCA cycle metabolites, such as α-ketoglutarate (α-KG). In addition to being a precursor for glutamine and glutamate, α-KG is a substrate for 2-oxoglutarate-dependent oxygenases (2OGXs), a key family of enzymes that participate in a variety of reactions leading to histone and nucleic acid demethylation and protein hydroxylation, among others [5]. In fact, reduced α-KG in Poldip2 deficient cells is sufficient to inhibit prolyl hydroxylases and drive HIF-1α stabilization even in normoxia [3].

Second, the accumulation of acetyl-CoA due to its inability to be oxidized through the TCA cycle. An increased concentration of acetyl-CoA may facilitate the synthesis of ketone bodies or steroids steroid hormones. In addition of being a key metabolite, acetyl-CoA is growingly recognized as an important signaling molecule [6]. Hence, it is not surprising that it may impact normal and oncogenic signal transduction, especially via protein acetylation. In particular, acetyl-CoA can modulate global levels of histone acetylation known to participate in regulating the promoters of genes involved in cell growth and division [7]. This may explain previous work linking Poldip2 expression to the regulation of the cell cycle [8] and the fact that exogenous expression of Poldip2 in cancer cells not only increases mitochondrial respiration but also represses cell growth [3].
